# Clinical and serological features of juvenile systemic lupus erythematosus in an italian tertiary centre of pediatric rheumatology

**DOI:** 10.1186/1546-0096-12-S1-P325

**Published:** 2014-09-17

**Authors:** Irene Pontikaki, Andrea Becciolini, Maurizio Gattinara, Maria Gerosa, Silvana Zeni, Pier Luigi Meroni, Valeria Gerloni

**Affiliations:** 1 Department of Rheumatology, Unit of Pediatric Rheumatology, Chair of Rheumatology, University of Milan, Italy; 2 Department of Rheumatology, Chair of Rheumatology, University of Milan, G. Pini Institute, Milan, Italy

## Introduction

Juvenile systemic lupus erythematosus (jSLE) is a multisystem autoimmune disorder that is characterized by widely variable clinical presentations and uncertain course.

## Objectives

to evaluate the clinical and serological features of jSLE in a long lasting observation of a single italian centre.

## Methods

104 patients affected by jSLE (< 18 years) were enrolled in thirty one years (from 1982 to 2013). 11 patients were male. The mean age of onset was 13 (SD 3.18); 41 were 12 years old or less. The mean disease duration was 163 months (SD 118).

## Results

Fever and fatigue were frequent symptoms at onset, occurring respetively in 47 (45.1%) and 39 (37.5%) patients. The most common organ involvement was skeletal, affecting 89 (85.5%) of patients, 87 (97.7%) of whom were affected by non erosive arthritis. Jaccoud's arthropathy was observed in 7 (6.7%) patients. Malar rash, leukopenia and non scarring alopecia were common findings. They occurred respectively in 60 (57.6%), 59 (56.7%) and 52 (50%) patients. Clinical evidence of renal involvement occurred in 42 (40.3%) patients. 31 patients had 1 or more renal biopsies: 22 (70.9%) resulted affected by diffuse proliferative glomerulonephritis, 6 (19,3%) by focal proliferative glomerulonephritis and 6 (19.3%) by membranous nephropathy. Cutaneous vasculitic lesions, observed in 38 (36.5%) patients, were an important cause of morbidity. Oral ulcers and serositis were found in 27 (25.9%) and 25 (24%) patients respectively. Neurologic involmentent occurred in 20 (19.2%) patients. Seizures were observed in 8 (7.6%) patients. Infections remain a major problem in morbidity: serious infective manifestations occurred in 21 (20.1%) patients. Avascular necrosis of bone occurred in 7 (6.7%) patients, 3 of whom suffered more than 1 episode. Six (5.7%) patients developed steroid induced cataract. Tendon ruptures were observed in 4 (3.8%) patients. Growth failure and established osteoporosis, resulting from prolonged corticosteroid treatment in chronically active disease, were unfrequent but severe complications in our jSLE series occurring respectively in 8 (7.6%) and 5 (4.8%) patients. Cushingoid features and striae rubrae were frequent problems observed in 17 (16.3%) patients, due to disease itself and/or its treatment, that caused increased psychological distress, particularly in adolescents. Finally there were 4 deaths. Two due to infection complications and two due to myocardial infarction.

## Conclusion

these data show that jSLE is not necessarily associated with poor prognosis; survival has improved, but morbidity due to disease itself and complications of therapy remain a significant problem.

## Disclosure of interest

None declared

